# Groin Hernia Surgery in Uganda: Caseloads and Practices at Hospitals Operating Within the Publicly Funded Healthcare Sector

**DOI:** 10.1007/s00268-020-05633-9

**Published:** 2020-06-15

**Authors:** Alphonsus Matovu, Pär Nordin, Andreas Wladis, Mary Margaret Ajiko, Jenny Löfgren

**Affiliations:** 1 Department of Molecular Medicine and Surgery, Karolinska Institutet, Karolinska University Hospital, 17176 Stockholm, Sweden; 2 Department of Surgery, Mubende Regional Referral Hospital, Plot M.4 Kakumiro Road, P.O Box 4, Mubende, Uganda; 3 Department of Surgery and Perioperative Sciences, Umeå University, Umeå, Sweden; 4 Department of Clinical and Experimental Medicine, Linköping University, Linköping, Sweden; 5 Department of Molecular Medicine and Surgery, Karolinska Institute, Solna, Sweden

## Abstract

**Background:**

Groin hernia is a major public health problem with over 200 million people affected. The unmet need for surgery is greatest in Sub-Saharan Africa where specialist surgeons are few. This study was carried out in Uganda to investigate caseloads and practices of groin hernia surgery at publicly funded hospitals.

**Methods:**

The study employed mixed methods covering 29 hospitals: the National Referral Hospital (NRH), 14 Regional Referral Hospitals (RRH) and 14 General Hospitals (GH). In part one of the study, surgeons and medical doctors performing hernia repair were interviewed about their practices and experiences of groin hernia surgery. In part two, operating theater records from 2013 to 2014 from the participating hospitals were reviewed and information about groin hernia operations collected.

**Results:**

All respondents reported that sutured repair was the first-choice method. A total of 5518 groin hernia repairs were performed at the participating hospitals, i.e., an annual hernia repair rate of 7/100 000 population. Of the patients operated, almost 16% were women and 24% were children. Local anesthesia (LA) was used in 40% of the cases, and non-surgeon physicians performed 70.3% of the groin hernia repairs.

**Conclusion:**

Groin hernia repair outputs need to increase along with the training of surgical providers in modern hernia repair methods. Methods and outcomes for hernia repair in women and children should be investigated to improve the quality of care.

## Introduction

Groin hernia repair is the most commonly performed general surgical procedure worldwide [1]. Over 200 million people are affected with groin hernias globally [2], and untreated groin hernias have significant morbidity and mortality [3]. In Sub-Saharan Africa (SSA), the volume of groin hernia surgery relative to the prevalence is insufficient [4–6]. Many people live with untreated groin hernias representing an enormous unmet need for surgery [7, 8]. In Ghana, the annual incidence of symptomatic hernias was 210/100,000 people with an estimated hernia repair rate of 30/100,000 [9]. In Uganda, the total groin hernia prevalence rate was 9.4%, 6.6% untreated or with recurrence and 3.3% with scars and an annual inguinal hernia repair rate of 17/100,000 people [7]. In Sierra Leone, of the 93.2% people who indicated the need for health care, only 22.3% underwent a surgical procedure [10].

Groin hernia surgery techniques have evolved from stimulating inflammation by cauterization and application of a golden stitch, using a hernia support, narrowing of the hernia orifice, the Bassini and Shouldice suture methods to the current tension-free mesh repair [11–13]. The Shouldice technique is superior to non-mesh based methods; however, it is usually performed by highly trained surgeons at specialized centers [14]. Over time, the use of synthetic mesh is recommended as the first-choice method in inguinal hernia repair, because of less recurrence and postoperative pain [15]. Further potential improvements in groin hernia surgery include use of Local Anesthesia (LA) and day case surgery [16–19]. The extent of mesh application, LA and day case surgery in SSA is not known. This nationwide study from 2013 to 2014 investigated surgical caseloads and practices of groin hernia surgery in Uganda, an East African low-income country of 39 million inhabitants [20].

## Materials and methods

**Fig. 1:**
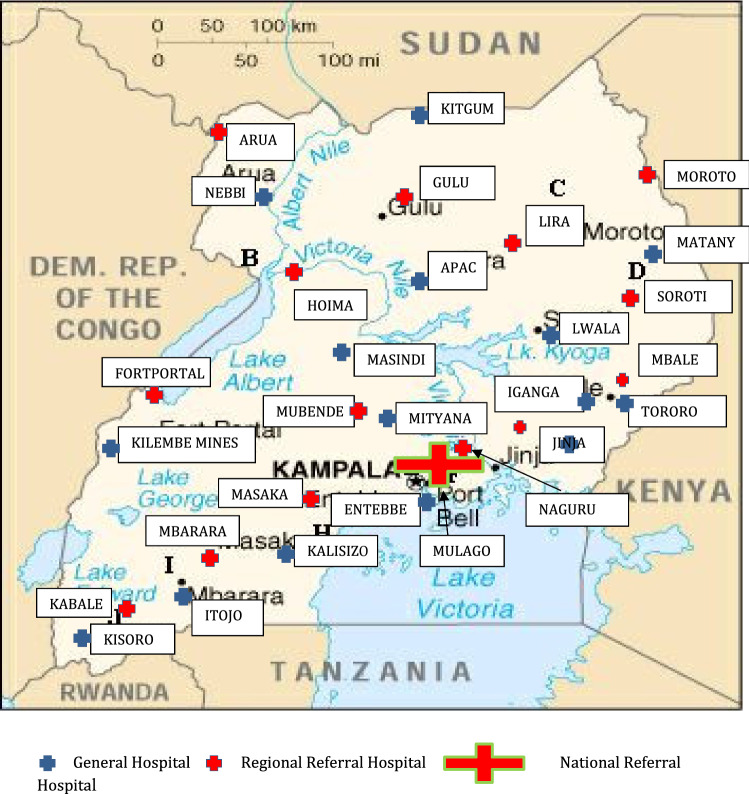
Geographic distribution of hospitals included in the study

This study was carried out at 29 publicly funded hospitals throughout Uganda (Fig. 1). These were the National Referral Hospital (NRH), all 14 Regional Referral Hospitals (RRH) and 14 General Hospitals (GH) each affiliated to a RRH. The GHs were selected according to convenience. In Uganda, the healthcare system has primary, secondary and tertiary levels. The GH represents the primary level covering a catchment population of approximately 500,000 people. Specialist surgeons and anesthesiologists are normally not employed in these hospitals where basic surgical procedures such as hernia repair and cesarean section are performed. The RRH represents the secondary level of healthcare, constructed for a catchment population of two million people. The services offered include specialized medical and surgical care, and at least one specialist surgeon should be employed. In addition, RRHs train intern doctors who can perform surgical procedures like hernia repair and cesarean section, medical, paramedical and nursing students. They support and receive patients from the GHs within their catchment area. The NRH is the tertiary level of healthcare serving the whole population with subspecialized medical and surgical care, advanced diagnostics, research, and training of medical doctors, nurses and paramedics [21].

The study comprised two parts. Part one was a questionnaire-based interview answered by surgeons and medical doctors performing groin hernia repair at the participating hospitals. The questionnaire was piloted by the lead investigator before using it in the study. From each hospital, a surgically active surgeon or medical officer was interviewed. A surgeon in Uganda is usually a medical doctor who has completed a minimum of 3 years of surgical training following medical school and internship. A medical doctor is a doctor who has completed medical school and internship.

The questionnaire included methods for hernia repair and anesthesia used in elective and emergency groin hernia surgery, duration of hospital stay and practice of day case surgery. Interviews were carried out by the research team after obtaining informed consent from the respondents.

Part two was a retrospective review of data from 2013 to 2014 theater records. These records are routinely kept at all hospitals in Uganda. Record books may be found stored in the operating theater or in the archives. The theater records have information on surgical volume, patient demographics and the procedures performed at any particular hospital. The information includes date of the surgical procedure, gender, age, patient diagnosis, the surgeon’s name, the surgical procedure and the form of anesthesia used. Data on groin hernia procedures were extracted from these records and entered into excel spread sheets. Some of the data were missing, and the recovered data were used to cater for missing data by imputation. Microsoft Excel and SPSS were used for descriptive and comparative data analyses.

Ethics approval was obtained from Makerere University School of Public Health Higher Degrees Research and the Ethics Committee and the Uganda National Council of Science and Technology. Prior to data collection, the Medical Superintendent and Hospital Director of each hospital signed a consent form authorizing collection of data. All respondents in part one of the study gave their informed consent prior to the interview.

## Results

Twenty-nine respondents, 16 surgeons and 13 medical doctors, were interviewed (Table 1). Sutured techniques, mainly the modified Bassini, were the methods of choice for all respondents in both elective and emergency groin hernia repair. The reasons mentioned were availability of sutures, affordability and that the technique is well known. The respondents rarely used the mesh technique due to non-availability of the mesh, high costs and lack of possibilities to learn how to perform mesh repair. Its use was limited to teaching and patients who could afford it.

**Table 1:** Interviews with key surgical providers in the 29 study hospitals

	Specialist (*n* = 16)^a^	Medical doctor (*n* = 13)	*P* value
*First choice for surgical technique*			
Sutured technique [*n* (%)]	16 (100)	13 (100)	1.0
*First-choice form of anesthesia, elective surgery*			
Local anesthesia	9 (56.3)	6 (46.1)	0.289
Spinal anesthesia	3 (18.8)	6 (46.1)	
General anesthesia	4 (25)	1 (7.7)	
*First-choice form of anesthesia, emergency surgery*			
Local anesthesia	2 (12.5)	0 (0)	0.168
Spinal anesthesia	3 (18.8)	6 (46.2)	
General anesthesia	11 (68.8)	7 (53.8)	
*Day case surgery*			
Yes	10 (62.5)	8 (61.5)	1.0
*Preferred length of hospital stay after elective surgery (days)*			
<1	3 (18.8)	2 (15.4)	1.0
1–3	10 (62.5)	10 (76.9)	
4–6	2 (12.5)	1 (7.7)	
7 or more	1 (6.3)	0 (0)	

^a^Fourteen specialist surgeons and two specialist gynecologists

In elective cases, 56.3% of the specialists and 46.1% of the medical doctors prefer LA in a small inguinal hernia and day case surgery. The mentioned benefits of using LA were affordability and not requiring anesthetic resources because surgeons administer it themselves. There was no distinction between LA and ilioinguinal nerve block. Spinal Anesthesia (SA) was the method of choice for patients with a large inguinoscrotal hernia, for recurrent hernia and when the absence of oxygen prevented the use of General Anesthesia (GA). SA could also be used instead of LA to avoid pain or discomfort associated with LA. GA was preferred by 25% of the specialists and 7.7% of the medical doctors mainly for large inguinoscrotal hernias and pediatric groin hernia surgery. Patient preference occasionally determined the choice for anesthesia.

In emergency cases, obstructed or strangulated groin hernia with suspected gangrenous bowel, 68.8% of the specialists and 53.8% of the medical doctors preferred GA. SA was the second most common form of anesthesia, especially for obstructed or strangulated groin hernia or when GA was not available. In emergency cases with small obstructed hernia and no suspicion of gangrenous bowel, LA was preferred by two of the respondents.

Day case surgery was practiced in 17 hospitals, mostly due to limited bed space and patient preference. Furthermore, the respondents stated that it is cost-effective for both the hospital and the patients. The other 12 respondents did not practice day case surgery due to the common use of GA or SA and long journeys for patients to return home. The respondents said that some patients preferred to be admitted until all sutures are removed, in other cases the operating doctor preferred to observe patients for at least 24 h prior to discharge so that early complications could be attended to. Duration of hospital stay varied between less than 1 day to more than 7 days, mainly depending on whether the procedure was performed electively or as an emergency.

**Fig. 2:**
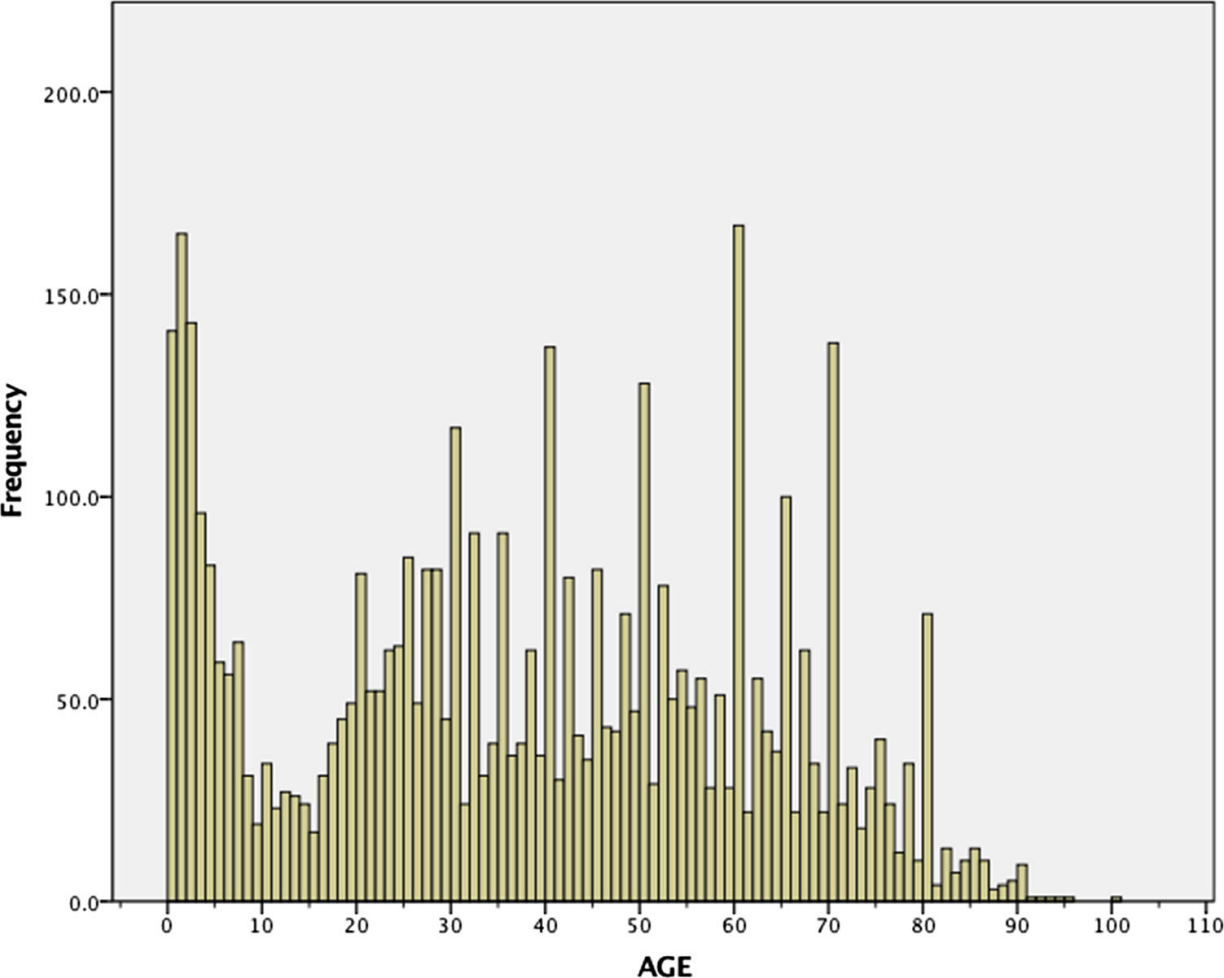
Age distribution of patients who had groin hernia surgery

Record reviews showed that 5518 groin hernia repairs were performed in the 29 hospitals in 2013 and 2014. Of the patients where gender was reported, women represented 15.9% (*n* = 762) of the patients and 23.3% of the patients were below 18 years. The age distribution was biphasic with a peak incidence of hernia repair in the age group under 5 years and a second peak later in life (Fig. 2).

The numbers of hernia repairs in 2013 and 2014 were almost identical: 2765 and 2753, respectively. The operations were mainly performed at the GH (*n* = 2430, 44%) and the RRH (*n* = 2711, 49.1%). Only a few procedures were performed at the NRH (*n* = 377, 6.8%). The Ugandan population was 39 million in 2014, making the collective surgical volume of the hospitals included in this study 7 hernia repairs per 100,000 population and year.

Medical doctors performed 2620 (50.8%) of the groin hernia repairs, while intern doctors performed 1006 (19.5%). The remaining repairs were performed by specialist surgeons (Table 2). Specialist surgeons together with intern doctors performed the majority of the hernia repairs in the RRHs, whereas medical doctors performed the majority of the repairs in the GHs and the NRH. In the RRHs and the NRH, the surgeon was mostly assisted by another medical doctor, while in the GHs the assistant was usually a nurse.

**Table 2:** Level of training of the surgeon, surgical and anesthesia methods at the National Referral Hospital, the Regional Referral Hospitals and the General Hospitals

	NRH [*n* (%)]	RRH [*n* (%)]	GH [*n* (%)]	*P* value
*Level of training of surgeon, n *= *5157*				
Specialist surgeon	52 (16.1)	1038 (39.4)^a^	77 (3.6)	<0.01
Medical doctor after internship	261 (81.1)	589 (22.4)	2125 (96.4)	
Intern doctor	9 (2.8)	1006 (38.2)	0 (0)	
*Level of training of assistant, n *= *3511*				
Medical doctor after internship	41 (17.8)	216 (10.1)	118 (10.2)	<0.01
Intern doctor	169 (73.5)	1585 (74.4)	0 (0)	
Nurse	15 (6.5)	167 (7.8)	700 (60.8)	
Student	4 (1.7)	123 (5.8)	37 (3.2)	
Theater assistant	1 (0.4)	38 (1.8)	297 (25.8)	
*Level of training of anesthetist giving spinal and general anesthesia, n *=* 2900*				
Specialist anesthesiologist	38 (14.3)	84 (6.1)	0 (0)	<0.01
Anesthetic officer	215 (80.8)	975 (70.9)	915 (72.7)	
Anesthetic assistant	1 (0.4)	140 (10.2)	203 (16.1)	
Surgeon	2 (0.8)	33 (2.4)	1 (0.1)	
Medical officer after internship	1 (0.4)	105 (7.6)	67 (5.3)	
Intern doctor	0 (0)	30 (2.2)	0 (0)	
Nurse	0 (0)	0 (0)	14 (1.1)	
Student	9 (3.4)	8 (0.6)	59 (4.7)	
*Anesthesia method, n *= *5010*				
Local anesthesia	41 (13.1)	1106 (43.7)	859 (39.6)	<0.01
Spinal anesthesia	81 (26.0)	511 (20.2)	436 (20.1)	
General anesthesia	190 (60.9)	912 (36.1)	874 (40.3)	

^a^Including three operations by a gynecologist

LA and GA were used to the same extent: 40.0% and 39.4%, respectively. SA was used in 20.5%. The most common healthcare professional administering GA and SA was the anesthetic officer. These are professionals with a 3-year diploma in clinical medicine, midwifery or nursing in addition to 2 years training in anesthesia. Specialist physician anesthetists administered 4.2% of the GA and SA.

## Discussion

The results of this study on hernia repair performed within the Ugandan publicly funded healthcare sector reveal that hernia repair is most often performed under local or general anesthesia using sutured techniques. The tertiary and the secondary hospitals together with a selection of GHs perform around 7 groin hernia repairs per 100,000 population and year. The operations are mostly performed by non-specialist medical doctors. Women represent 16% of the cases, and almost a quarter are children.

To improve the quality of surgery, hernia repair techniques must comply with international standards. In this study, all respondents use sutured techniques as their first choice. This means that majority of patients may not benefit from the advantages of mesh repair with lower recurrence rates compared to sutured methods [22]. Sutured techniques are preferred to mesh repair because mesh technique is expensive, not known or mastered by the respondents.

Surgical safety is a particular concern and increased use of LA would be an important step in that direction. LA is recommended for uncomplicated, unilateral, open primary hernia repair [23, 24]. It is safe, effective and more cost-effective than SA or GA [25, 26]. In this study, LA was used in 40% of hernia repairs. This is far higher than the 1.1% in another SSA setting [27]. It is also far higher than in high-income countries like Sweden where only 13% of groin hernia repairs are performed under LA. If LA is used, the anesthesia workforce can direct their attention to procedures that require SA or GA.

The groin hernia surgery rate demonstrated in this study is low considering that these hospitals represent the largest public hospitals in the country. Hernia repair has been found to be the most commonly performed general surgical procedure in previous studies in the region [4, 6]. Still, the prevalence of untreated groin hernia was above 6% in adult men in a study from Eastern Uganda [6]. Increasing the number of repairs is essential to reduce the burden of disease due to hernia. In Ghana, a country with a large unmet need for groin hernia repair, it is estimated that an operation rate of 420 per 100,000 inhabitants per year would eliminate symptomatic groin hernia within 10 years [5, 9]. This elimination repair rate was developed by equating the inguinal hernia prevalence, to the symptomatic inguinal hernia incidence, the symptomatic inguinal hernia incidence, surgical repair, the 100-year case backlog and the DALYs associated with backlog in millions [9]. This rate is higher than in western countries (130–290/100,000 population and year) where backlog is minimal [28, 29]. Our methodology was not set to determine the operation rate that would eliminate the backlog in Uganda, but it would probably require a similar or higher target rate to that in Ghana.

The majority of hernia repairs were performed by medical doctors, while interns performed close to 20% of all hernia repairs. Task-sharing involves the delegation of certain medical responsibilities to less specialized health care workers [30, 31]. In Uganda, specialist and resident surgeons are few in number and unevenly distributed between urban and rural areas. Basic surgical procedures such as groin hernia repair are therefore frequently performed by non-specialist surgeons. The provision of structured training for intern doctors and non-specialist physicians would increase surgical capacity and to improve the quality of surgical care for hernia in Uganda. In order to build such capacity, hernia repair must be advocated for as a public health intervention. A hernia policy must include clinical guidelines, availability of LA and inexpensive, high-quality surgical mesh. A system for monitoring and evaluation of the quality of the surgical services delivered is also needed.

Almost 16% of the patients operated for groin hernia were female which is less than the 24% demonstrated in a recent but smaller study in Eastern Uganda, but more than twice the figures from other countries in Sub-Saharan Africa and Sweden [6, 23, 32, 33]. This may be a result of a higher prevalence of groin hernia in Ugandan women compared to elsewhere, or differences in health seeking behavior. Further on, almost a quarter of the patients were children. Due to the high risk of incarceration in women and children, groin hernia is an absolute indication for surgery.

The strength of this study is that it is based on interviews with surgeons and on review of theater records from primary, secondary and tertiary hospitals throughout Uganda. This gives high internal validity. The weaknesses are as follows: not all GHs and lower level healthcare units where hernia repair is performed were included in the study. This was due to limited funding and time. Accessibility and quality of data in the theater records was an issue of concern in this study. Handwritten records are difficult to read and deteriorate over time. In some instances, hernia data were missing due to destruction or loss of records. The available data were imputed to cater for the missing data. Processing those records into meaningful information is time consuming. The study was carried out for the years 2013 and 2014. There is no groin hernia surgery initiative so far in Uganda, and hernia mesh is still not widely available. It is expected that the situation has not changed much since 2013. Outcomes of the groin hernia operations are one of the indicators for quality of care but were beyond the scope of this study.

Future research should focus on how to design large-scale hernia surgery training initiatives and determining the epidemiology of groin hernia in women and children so that surgical services can be adapted to meet the specific needs of these groups. A hernia registry would facilitate the improvement transition and enable the making of timely reports and high-quality research in the future.
